# Predictions of Cu toxicity in three aquatic species using bioavailability tools in four Swedish soft freshwaters

**DOI:** 10.1186/s12302-015-0058-1

**Published:** 2015-10-12

**Authors:** S. Hoppe, M. Sundbom, H. Borg, M. Breitholtz

**Affiliations:** Department of Environmental Science and Analytical Chemistry (ACES), Stockholm University, 106 91 Stockholm, Sweden

**Keywords:** Copper, Soft freshwater, Sweden, Bioavailability tools, DOC, Al, Fe

## Abstract

**Background:**

The EU member countries are currently implementing the Water Framework Directive to promote better water quality and overview of their waters. The directive recommends the usage of bioavailability tools, such as biotic ligand models (BLM), for setting environmental quality standards (EQS) for metals. These models are mainly calibrated towards a water chemistry found in the south central parts of Europe. However, freshwater chemistry in Scandinavia often has higher levels of DOC (dissolved organic carbon), Fe and Al combined with low pH compared to the central parts of Europe. In this study, copper (Cu) toxicities derived by two different BLM software were compared to bioassay-derived toxicity for *Pseudokirchneriella subcapitata, Daphnia magna* and *D. pulex* in four Swedish soft water lakes.

**Results:**

A significant under- and over prediction between measured and BLM calculated toxicity was found; for *P. subcapitata* in three of the four lakes and for the daphnids in two of the four lakes. The bioassay toxicity showed the strongest relationship with Fe concentrations and DOC. Furthermore, DOC was the best predictor of BLM results, manifested as positive relationships with calculated LC_50_ and NOEC for *P. subcapitata* and *D. magna*, respectively.

**Conclusion:**

Results from this study indicate that the two investigated BLM softwares have difficulties calculating Cu toxicity, foremost concerning the algae. The analyses made suggest that there are different chemical properties affecting the calculated toxicity as compared to the measured toxicity. We recommend that tests including Al, Fe and DOC properties as BLM input parameters should be conducted. This to observe if a better consensus between calculated and measured toxicity can be established.

## Background

When implementing water quality criteria (WQC) for metals in aquatic environments it is crucial to consider water chemistry parameters such as hardness, concentration of humic substances and pH, since these strongly affect the speciation of many metals [1–4]. For instance, cations (e.g. Ca^2+^ and Mg^2+^) compete with other free metal ions (M^n+^) for biotic ligands, such as gill membranes, thereby reducing their uptake [2, 5, 6]. This protective effect is less pronounced in soft than in hard waters due to lower Ca^2+^ levels [7]. Total and dissolved organic carbon (TOC/DOC), especially the humic fractions, generally reduce metal toxicity by binding M^n+^ into less bioavailable, high molecular weight complexes [5, 6, 8]. The interaction of metals and organic carbon is also dependent on ambient pH, as hydrogen ions can compete with the M^n+^ for binding sites, presenting further challenges for accurately predicting the toxicity of metals in soft acidic freshwaters. In the process of setting WQC it is central to try to mimic organism exposure in natural environments [9], which can be difficult, given the vast natural variability within and among natural aquatic ecosystems. To facilitate this process, Biotic ligand models (BLMs) using freshwater chemical characteristics and chemical equilibrium calculations combined with organism biology can be used to calculate site-specific predicted no-effect concentrations (PNEC), environmental quality standards (EQS) and effect concentrations (LC/EC_50_) [6, 9–12]. For a reliable use in environmental risk assessment, BLMs use a variety of physico-chemical parameters, of which TOC, alkalinity and pH are the three most important for assessing metal toxicity [1, 13, 14]. In current BLMs, the calibration range is targeted towards the majority of south central European freshwaters [15]. However, in Scandinavia, freshwaters often have low levels of Ca^2+^ due to the dominating soft water qualities, combined with low pH values and high levels of DOC, Fe and Al [16]. According to Swedish national lake surveys, the median Ca^2+^ concentration is around 2 mg/L and the 90 percentile, 10 mg/L (*n* = 56,000), which consequently can be characterised as soft- to ultra-soft waters, often leaving them outside of the BLM calibration range [15, 17]. Studies have shown that chronic BLM-predicted Cu and Zn toxicity can be underestimated in freshwaters with low pH and elevated levels of TOC, Al and Fe [18, 19], a water quality commonly found in Sweden.

We hypothesize that the usage of BLMs outside of their targeted calibration range will also result in less reliable results compared to bioassay studies considering acute toxicity. To test this hypothesis, Cu toxicity was measured for three aquatic species: *Daphnia magna, D. pulex* and *Pseudokirchneriella subcapitata,* in water from four soft water lakes with different water chemistry. These lakes were chosen as representatives from the Swedish national monitoring program as they together represent the four most common Swedish water types (Älgsjön: dark humic forest lake, St. Envättern: uncontaminated lake with relatively high TOC, Fiolen: nutrient rich forest lake and Abiskojaure: nutrient poor clear water lake with low primary production). Results from the bioassays were compared with BLM-derived toxicities for chosen organisms and waters to see if there were any divergences between measured and calculated results.

## Results

### Chemical properties

**Fig. 1:**
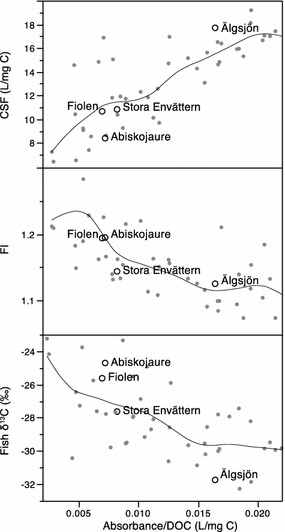
DOC properties: stable carbon isotope signatures (δ13C, multiannual mean) in perch muscle tissue, water fluorescence index (FI) and carbon specific fluorescence (CSF-) in Swedish lakes. The four lakes used in this study highlighted as *circles*

Table 1 presents the most important chemical characteristics of the four studied lakes, including Ca, Mg, Na, K, SO_4_, Cl, alkalinity (multi annual levels), pH, DOC, hardness as well as total dissolved (<0.2 µm) concentrations of Cu, Fe, Cd, Zn, Pb and Al sampled at the test start. The variability in pH, alkalinity and hardness is quite small among the lakes but they differ widely in DOC (0.9–17 mg/L), Fe (2–393 µg/L) and Al (1.5–57 µg/L) concentrations. Direct optical and integrated isotopic measurements suggest that the lakes also differ in the quality and origin of DOC. The four lakes fall within the typical ranges of Swedish lakes for these auxiliary optical and isotopic variables (Fig. 1). Älgsjön, a humic forest lake, displays not only the highest concentrations of DOC, Fe and Al, but also deviates most from the other three lakes in the ratio between absorbance (420 nm, 5 cm) and DOC, a measure of the carbon-specific colour of dissolved organic matter, and carbon-specific fluorescence (CSF), a measure of the relative fluorophore abundance, suggesting a different chemical composition of DOC in this lake as compared with the others. The fluorescence index (FI) and fish δ^13^C, indicative of the organic precursors of DOC, also varied among the lakes. The patterns indicate a higher portion of terrestrial carbon sources in Älgsjön compared to St. Envättern and especially to Fiolen and Abiskojaure (Fig. 1).

**Table 1:** Properties of the lake waters used in this study: lake pH and DOC concentrations were determined in the test water used for the bioassays

Lake	Abiskojaure (*n* = 32)	Fiolen (*n* = 58)	St. Envättern (*n* = 56)	Älgsjön (*n* = 58)
RT90 *X*–*Y*	758,208–161,749	633,025–142,267	655,587–158,869	655,275–153,234
Latitude	68.3067°N	57.0917°N	59.0948°N	59.0948°N
Longitude	18.6550°E	14.5317°E	16.3693°E	16.3693°E
Catchment	Above treeline, tundra vegetation	Coniferous forest, some agriculture	Coniferous forest	Coniferous forest, some wetlands
Lake area (km^2^)	2.8	1.6	3.7	0.35
Max depth (m)	35	10	11	7.7
pH^a^	7.6	6.5	6.5	6.5
DOC (mg/L)^a^	0.88	7.0	10	17
Ca (mg/L)	4.5 ± 1.46	2.9 ± 0.22	3.4 ± 0.19	5.6 ± 0.93
Mg (mg/L)	0.70 ± 0.21	1.0 ± 0.1	0.85 ± 0.06	1.9 ± 0.30
Na (mg/L)	1.0 ± 0.53	3.9 ± 0.27	2.2 ± 0.09	3.2 ± 0.54
K (mg/L)	0.59 ± 0.13	1.5 ± 0.15	0.29 ± 0.02	0.85 ± 0.15
SO_4_ (mg/L)	4.3 ± 1.35	6.2 ± 0.68	5.9 ± 0.56	5.6 ± 1.31
Cl (mg/L)	1.1 ± 0.87	6.0 ± 0.51	2.8 ± 0.17	2.8 ± 0.42
Alk (mg CaCO_3_/L)	10 ± 4.43	3.0 ± 0.76	3.0 ± 0.62	13 ± 3.31
Hardness (mg CaCO_3_/L)	13	9.8	10	18
Cu (µg/L)*	0.91	0.74	0.38	0.78
Fe (µg/L)^a^	2.01	33	31	393
Cd (µg/L)^a^	0.012	0.034	0.006	0.006
Zn (µg/L)^a^	0.5	5	1.6	0.8
Pb (µg/L)^a^	0.005	0.1	0.06	0.08
Al (µg/L)^a^	1.5	49.7	33.7	56.9

Major ion concentrations represent multi-annual means of all data 2000–2009 from the Swedish national monitoring program. Trace metal concentrations (0.22 µm filtered) were determined in the *Daphnia* test waters at test 0 and 48 h, the numbers presented is at test 48 h

* Original concentration without any addition, detection (± 0.08)

^a^Analysed from bioassays at the end of 48 h

### BLM calculations vs. bioassays

Both calculated and bioassay *Daphnia* LC_50_ values were lowest and highest in Abiskojaure and Älgsjön, respectively. The same was noticed for the algae concerning calculated NOECs and bioassay EC_50_ values (Table 2). These two lakes also differed most with respect to DOC, Fe and Al among the four investigated lakes in this study. BLM calculations resulted in higher LC_50_ compared to the measured (bioassays) ones in three of the four lakes for daphnids (bioassays with *D. pulex* was only available for two lakes due to cultivating problems), and higher BLM NOEC compared to measured EC_50_ for *P. subcapitata* in all four lakes (Table 2). The difference between calculated and measured toxicity for *D. magna* was statistically significant for Abiskojaure (*p* = 0.025) and Älgsjön (*p* < 0.001), however, differing only by a factor 2 and 1.3, respectively. For the alga, the difference was ranging from a factor 2.5 in Älgsjön to 9.3 in St. Envättern, proving to be significant in three of the four lakes (*p* < 0.001). The only exception was Älgsjön (*p* = 0.365) where BLM suggested higher toxicity to *D. magna* than the bioassays. Calculated BLM LC_50_ values for Abiskojaure and St. Envättern were high enough too corresponded to bioassay LC_100_ (LC_99_*D. magna* Abisko: 13.2 µg Cu/L; St. Envättern: 59.5 µg Cu/L) rather than a 50 % mortality. In contrast, for Älgsjön, the calculated LC_50_ value was below the measured value with a factor of 0.7. BLM calculated PNEC values (Table 2) based on the HC_5_ concentration varied between 4.9 µg Cu/L (Abiskojaure) and 31.4 µg Cu/L (Älgsjön). For two of the four lakes, Abiskojaure and St. Envättern, the PNEC value was on par with or exceeded the measured algal EC_50_ value for growth inhibition.

**Table 2:** Test and modelled results: bioassay (mean and SD values) and BLM results (µg Cu/L) for the crustacean and algae species compared as well as the eventual significance (*p* value)

	Abiskojaure	Fiolen	St. Envättern	Älgsjön
*D. magna*				
LC_50_	8.53 ± 1.16	34.9 ± 1.78	34.3 ± 2.67	128 ± 23
BLM LC_50_	17.4	40.0	61.7	92.5
*p* value LC50/BLM	0.025*	1.0	0.48	<0.001*
*D. pulex*				
LC_50_	7.6 ± 1	30.4 ± 2	–	–
BLM LC_50_	10.2	19.6	30.3	45.3
*p* value LC50/BLM^a^				
*P. subcapitata*				
EC_50_	1.4 ± 0.2	27.4 ± 2.5	20.8 ± 0.4	111 ± 8.7
BLM NOEC	9.8	127	185	287
*p* value EC50/BLM	<0.001*	<0.001*	<0.001*	0.365
PNEC	4.9	14.5	20.6	31.4

As the BLM used for the algae only can produce NOEC values this was compared to the bioassay EC_50_ value

* Denotes a statistical significant difference (*p* < 0.05); – was not tested

^a^Was not tested due to lack of statistic material

### Influence by water chemistry

**Fig. 2:**
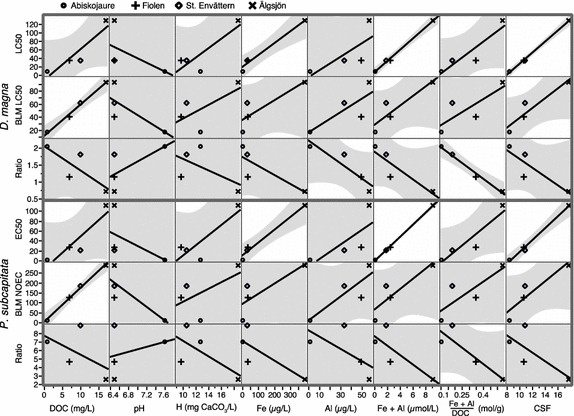
Water chemistry variables vs. BLM results: scatter-plot matrix of some water chemistry variables versus measured and BLM-derived toxicity indices for two species in four lakes. The *third* and *sixth rows* show the ratios between the* two rows* above. The *lines* are fitted linear regression lines and the *shadowed areas* represent the 95 % confidence interval of the* fitted line*

**Fig. 3:**
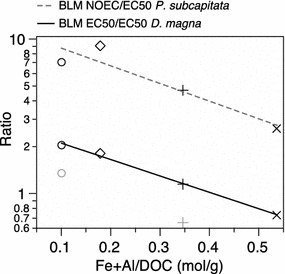
ANCOVA results for BLM vs. test results: Lines fitted by ANCOVA. The *lines* depict the relationship for the ratio between measured and BLM calculated toxicity (measured LC_50_, EC_50_) as well as the ratio between the molar sum of metals (Fe, Al) and DOC in four lakes. *Red line* ratio of LC50’s for *D. magna*; *blue line* ratio between NOEC and EC50 for *P. subcapitata*. The *greyed-out symbols* indicate LC-ratios for *D. pulex* that was exposed only to water from two lakes. *D. pulex* data was not part of the ANCOVA. The effect (slope) is statistically significant but the slopes does not significantly differ between the two species (Table 3)

Although BLM yielded higher values than bioassays, in 8 of 10 cases, the general direction of the regression slopes was coherent between BLM and bioassay estimates for the key chemistry variables. In an attempt to find which factors that could explain the observed systematic difference, the relationship between water chemistry and the ratios between BLM and bioassay results was plotted. The difference between BLM and bioassays was related to the ratio of dissolved metals and DOC, e.g. the ratio between Fe and Al and DOC (Figs. 2, 3). The correlation between estimated toxicity and all available variables was examined and a subset of these is visualized in Fig. 2. DOC was the best predictor of BLM calculated toxicity, whereas Fe, followed by DOC was the best predictor of bioassay results. Other key water chemistry parameters, such as pH and hardness, showed no clear relationship with toxicity for the tested waters. Compound and optical variables was also investigated and it was found that the molar sum of Al and Fe was a better predictor of bioassay toxicity for both test species than Fe was alone and CSF appeared to correlate strongly with bioassay toxicity (Fig. 2).

## Discussion

### Usage of BLMs in soft freshwaters

In this study, we applied two currently available BLM on four soft water lakes, over a wide range of DOC (0.8–17 mg C/L), Al and Fe concentrations in order to define Cu toxicity in typical Swedish freshwaters. The performance of these BLM software versions for waters outside of the intended calibration range was examined by comparing model output to measured acute Cu toxicity for *P. subcapitata, D. magna* and *D. pulex* (species only tested in two lakes). The *Daphnia* results showed that BLM v.2.2.3 both significantly over- and under-predicted Cu toxicity. In the lake where BLM overestimated toxicity, i.e. lake Älgsjön, a higher proportion of allochtonous natural organic matter (NOM), as compared to the other three lakes, was found, which indicates that lakes with these NOM properties could be less sensitive to Cu toxicity than lakes with a more autochthonous carbon source. However, for this model, both the over- and underestimation for the daphnids was within a factor of two, which could be considered a fairly good agreement. As for the algae, BLM v.0.0.0.17, underestimated the toxicity with a factor of 2.5 to 9.3, being significant in three out of four lakes (Table 2).

The lakes used in this study represent four typical Swedish freshwater lakes: *Älgsjön*: Dark humic forest lake with a high TOC, some months over 30 mg C/L, *St. Envättern*: uncontaminated lake with relatively high TOC, *Fiolen*: Nutrient rich forest lake; *Abiskojaure*: nutrient poor clear lake with low TOC and primary production. The lakes are used as reference lakes in the Swedish national monitoring program and are not considered to be affected by any local anthropogenic activities in their catchments, apart from some forestry. The only influence comes from long-range airborne pollutants. The levels of toxic trace metals (Pb, Cd, Cu, Zn) in the waters are low (Table 1), and should not affect the test organisms or compete with the added Cu for humic or biotic ligands. Concentrations of inorganic Al forms which are known to cause toxic effects on fish and invertebrates [20, 21] were found to be very low (<3 µg/L). This indicates that it is unlikely that toxic Al fractions contributed to any direct negative effects in this study.

### Differences between the models

The NOEC-values (BLM v.0.0.0.17) exceeded the bioassay EC_50_-values for the algae, with a factor of 2.5–9.3. They also exceeded the calculated PNEC-values by a factor of 2.2 in Abiskojaure up to a factor of 9.1 in Älgsjön and St. Envättern. Since the Cu NOEC value reflects the concentration where there should be no negative effect on the algal growth rate, it is notable that the NOEC value was higher than the measured EC_50_, i.e. the estimated Cu concentration for 50 % growth reduction. However, since the use of NOEC values in ecotoxicology is strongly questioned by international standardisations bodies [22], the BLM software should use an EC_x_ endpoint instead. BLM v.0.0.0.17 overall gave a larger deviation between calculated and measured values than BLM v.2.2.3. BLM v.2.2.3, which uses a conversion factor to calculate chronic toxicity, produced results for the two *Daphnia* species within a factor of two. Thus, in these soft waters BLM v.2.2.3 calculated more similar results compared to the bioassays. This since BLM v.0.0.0.17 calculated algae NOEC values (where no growth inhibition is expected), which were higher than the measured EC_50_ values (concentration where there was a 50 % growth inhibition). Furthermore, even though BLM v.2.2.3 is an acute model, the approach of using the conversion factor seems to be working, at least for the investigated waters.

### Influences by DOC, Fe and Al on toxicity

The soft water lakes used for this study covered a wide range in DOC, whereas other key variables such as pH and hardness were rather comparable among the lakes. As the hardness and alkalinity were similar there was no clear difference in the BLM performance to predict Cu-toxicity that could be related to the variation in these variables (Fig. 2). It is apparent that calculated effect concentrations derived by using these BLMs agree better with the measured effect concentrations at elevated Al+Fe/DOC-ratios. In other words, these BLMs or more specifically the speciation model WHAM V (Windermere Humic Acid model) which is incorporated into BLM 2.2.3, will produce more accurate calculations of metal speciation when there is a higher amount of metal ions that can bind to the NOM ligands present in the water. This has previously been shown [23] for both Cu and Cd, although, the problem was more pronounced for Cu. This relationship was also statistically significant when Fe was included and the *p* values decreased as more metals were added (Table 3). Up to a certain degree, the more metals per unit carbon that is present in the water, the better the agreement is between the bioassay and BLM results. An analogous relationship has previously been reported by Tipping [23, 24] who found that earlier versions of the WHAM V, which is used in BLM 2.2.3, calculated reliable simulations for copper titrations of humic and fulvic acids when the [Cu]/DOC ratios were high, but performed less well when the [Cu]/DOC ratio was low.

**Table 3:** ANCOVA results: ANCOVA table testing the effect of the ratios between metals and DOC (independent covariate) on the log-transformed ratios between toxicity indices estimated by BLM and bioassays (dependent variable) for two species *D. magna* and *P. subcapitata* (nominal factors)

	Al/TOC	Fe/TOC	∑FeAl/TOC (mol/g)	∑Metals/TOC (mol/g)
*R* ^*2*^	0.739	0.945	0.980	0.985
Root mean square error	0.590	0.270	0.163	0.142
Analysis of variance				
*F*_3.7_ ratio	3.77	23.04	65.59	87.37
Prob > *F*	0.1164	0.0055	0.0007	0.0004
Effect tests:				
Species	0.03	0.002	0.0003	0.0002
log(Me/TOC)	0.5493	0.0144	0.0018	0.0010
Species *x* log(Me/TOC)	0.93	0.79	0.80	0.72
Estimated slope of covariate ± SE				
log(Me/TOC)	−0.069 ± 0.11	−0.046 ± 0.01	−2.531 ± 0.34	−2.655 ± 0.31

See Fig. 3 for further details

#### DOC properties

The important aspect of the dissolved organic matter (DOM) quality is not considered in the two BLM softwares used in this study. This could be an important factor as DOM properties have been shown to influence both speciation [23–29] and toxicity [30] of Cu. Älgsjön, where BLM calculations overestimated the Cu toxicity compared to bioassays, differs from the other lakes concerning the ratio between water colour (absorbance of filtered water at 420 nm) and DOC. This water colour ratio was found to be similar among three of the lakes, 0.007, 0.008 and 0.009 in Abiskojaure, Fiolen, and St Envättern, respectively, whereas Älgsjön had a higher ratio of 0.018 (Fig. 1). The latter value, to some extent, likely reflects the higher relative Fe concentration in this lake. However, spectrofluorometric and isotopic data also suggest that there are qualitative differences in the DOC among the lakes which can influence the DOC complexing capacity for trace metals. In particular, the CSF showed a correlation with Cu toxicity. As a measure of the fluorophore density, CSF increase with the degree of aromatization which at current wavelength settings is indicative of humic substances. Fiolen and St. Envättern had very similar CSF as well as toxicity indices derived by both BLM and bioassays, while Fiolen in terms of C origin instead was very close to Abiskojaure, the lake most sensitive to Cu. Fiolen and Abiskojaure were estimated to have a more autochthonous DOC than St. Envättern and especially Älgsjön were characterized by allochtonous DOC (Fig. 1). Within the narrow ranges of the other parameters no clear linear patterns could be seen.

#### Fe and Al

In the bioassays, varying concentrations of Fe and Al may influence the toxicity of Cu, while BLM-calculated toxicity will not be affected, as these metals are not among the model input parameters. For this reason, it is not surprising that the BLM estimates were best correlated with DOC, as DOC supposedly is one of the most important input variables in current BLM versions. However, bioassay results suggested that Fe, especially in combination with Al, is as important as DOC for the Cu toxicity to the tested organisms (Fig. 2). An additional explanation for the overestimation of toxicity in Älgsjön could be connected to the high Fe and DOC content in the lake, creating humus-iron aggregates as well as inorganic colloidal and particulate Fe-forms, such as ferrihydrite, which are fairly adsorptive for other trace metals, including Cu [31]. Even though the water was filtrated, aggregates could have been formed during the time between the filtration and the test start. These strong adsorbents may influence the Cu-speciation and could contribute to a decreased concentration of free Cu-ions in the dissolved bioavailable phase, which then would decrease the toxicity.

When comparing calculated with measured results, the magnitude and direction of the plotted slopes (Figs. 2, 3) were consistent for both the daphnids and the alga, suggesting that the bioavailability of Cu to these organisms is regulated by the same mechanisms. The degree of underestimation, defined as the ratio between BLM and measured toxicity was, for both the alga and the daphnids, best correlated with the molar ratio between the sum of metals (Fe and Al) and DOC (Fig. 2**)**. As Fe had the strongest correlation when single variables were considered, Fe was in this case apparently the more important of the two (Fig. 2). The slope patterns are similar for the tested species, as shown by the ANCOVA where the interaction term was non-significant (Fig. 3; Table 3). It has previously been shown [24] that both Al and Fe can compete with Cu for binding sites at the DOC, especially in cases with low Cu levels, affecting the speciation in the water.

A few other studies have indicated that BLM could underestimate Cu toxicity for *P. subcapitata* (factor of 2–4), chronic toxicity for *D. magna* (factor 8) and toxicity to larval fathead minnows in soft waters [8, 14, 18, 19, 32]. Some of these studies suggest that the underestimation by BLM on Cu toxicity could be explained by the model not sufficiently accounting for Fe or Al. Indicating that these metals are competing with Cu for the binding sites on humic substances, preventing Cu from binding to the humic ligands and instead increase the bioavailability.

## Conclusion

This study shows that using current BLM software (i.e. v.0.0.0.17 and v.2.2.3) outside of their intended calibration range could prove problematic since the models differed in their ability to accurately predict toxicity. The significant difference between measured and calculated toxicity was found to be between a factor of 0.7 up to 9.3. The toxicity measured in the bioassays showed the strongest relationship with concentrations of Fe, Al and DOC. In the lake where BLM overestimated Cu toxicity, different properties as regard to DOC origin was found, indicating that qualitative DOC properties can affect the performance of the BLM calculations. As our results are indicative towards Al, Fe and NOM properties influencing BLMs ability to calculate Cu toxicity we strongly recommend that studies considering the implementation of these parameters are conducted. DOM composition is extremely complex and not easily implemented in operative models like BLM; still, if the important aspects of DOC can be condensed into simple and cost effective optical properties like carbon-specific fluorescence or absorbance, as our results indicate, it could be worthwhile to further investigate.

## Methods

### Sampling and treatment

The selected lakes; Abiskojaure, Fiolen, St. Envättern and Älgsjön are included in the Swedish national monitoring program, sampled 8–10 times/year for the last 20 years, and are consequently well characterized both chemically and biologically. The lakes vary regarding chemistry as well as catchment properties (Table 1). All of the lakes have low hardness (9–19 mg CaCO_3_/L) and circumneutral pH (6.5–7.6), and differ widely in TOC (0.8–17 mg/L). The water used in this study was sampled 2011. The pH values of these lakes were kept in the neutral range. Water from Abiskojaure was collected by staff at Abisko Scientific Research station (Royal Swedish Academy of Science), using polycarbonate water sampler from the ice, in March–April, and sent to ITM (Dept. of Applied Environmental Science). Water from Fiolen was similarly collected by staff at the County Administrative board in Växjö and sent to ITM. These two waters were filtered upon arrival, in a clean room, within 3 days after sampling. Water from Älgsjön and St. Envättern were sampled by the authors and filtered in situ or the next day in a clean room. To avoid contamination of test waters, acid cleaned (0.1 M HCl) 5 L polypropylene containers were used. All filtrations were performed on-line by pumping water through acid cleaned (0.1 M HCl) plastic tubes (Masterflex tubing, silicone) and 0.22 µm acid cleaned (0.1 M HCl) polypropylene capsule filters (Calyx capsule). The filtered water was placed in a dark cold room at 6 °C for 1 month until time of analysis and bioassay testing.

### Water chemical analyses

Physico-chemical measurements were performed by the Swedish University of Agricultural Science (SLU) (major ions), ACES (trace metals, O_2_, pH and fluorescence) and Stockholm Water Company (Stockholm Vatten) (TOC). Oxygen saturation levels and pH were determined using a SympHonic SP90N5 multimeter and a Radiometer pHM82 Standard. Trace metal levels were analysed both at the start of the bioassays and after 48 h using an ICP-MS Thermo X-series II (inductively coupled plasma mass spectrometry). The ICP-MS accuracy was checked using certified reference water (SLRS-4 riverine water; Cu: certified 1.81 ± 0.08 µg/L) during each test batch. The labile inorganic Al fraction in the lake waters was determined by cation exchange spectrophotometry [33]. TOC was determined using a Shimadzu TOC-5050, and DOC was assumed to be 90 % of TOC concentrations. DOC is often considered to differ approximately 5 % from TOC [34, 35], however, since there could be aggregates present, due to the water standing for one month prior to test start, 10 % was chosen. Fluorescence emission spectra of the lake waters were determined by a spectrofluorometer (JASCO FP-777) equipped with a 150 W xenon lamp and monochromator. Excitation wavelength was set to 370 nm and intensities were corrected for inner filtering effects and blanks. The fluorescence spectra were used to determine the carbon-specific fluorescence (CSF, ratio between max fluorescence and TOC concentration) and the fluorescence index (FI, the ratio between intensities at 450 and 500 nm) [36]. Both CSF and FI are optical measures of DOC properties that can give e.g. the aromaticity and origin of dissolved organic matter [34]. Stable carbon isotope signatures (δ^13^C) in fish (Sundbom, unpublished data) were measured by the Stable Isotope Facility, University of California at Davis, using isotope ratio mass spectrometry (PDZ Europa 20-20, Sercon Ltd., Cheshire, UK). This measurement has previously been shown to provide an indication of the amount of allochtonous carbon found in the water, meaning that at a higher δ^13^C (more negative) the DOC often is of allochtonous origin. All chemical analyses were conducted following quality assurance routines specified in the accreditation of the laboratories.

### Bioassays

#### Daphnia spp

*Daphnia* tests were conducted using both *D. magna* and *D. pulex*. The *D. magna* clone was provided by University of Göteborg in Sweden, where it has been cultured since 1979. The clone originated from a small lake in Bohuslän in the south west of Sweden. The *D. pulex* clone (PA_33_: Portland arch) was provided by University of Indiana, USA. Daphnids were cultured in groups of 15-20 animals in 3 L clear glass beakers containing 2.5 L of M7 medium. The M7 medium was renewed on a weekly basis and the animals were fed a mixture of *Monoraphidium contortum* (800 µL) and *P. subcapitata* (6.8 mL), approximately 0.2 mg C/day/*Daphnia*. The light cycle was 16:8 h light/darkness. The condition of the *Daphnia* culture was tested with K_2_Cr_2_O_7_ in synthetic soft water media (M7) and the LC_50_ found to be within the recommended range [37]. One month before test start, the M7 culture medium was exchanged to lake water in order for the daphnids to acclimatize. After one month the new cultures were in good shape and no visible differences compared to the mother culture were observed, except for Abiskojaure where the animals were slightly longer and thinner. Experimental setup was designed according to OECD Test Guideline No. 202 [38]. Briefly, neonates (<24 h) were exposed to five different concentrations of copper dissolved in 50 mL lake water; Abiskojaure: 0;4.6; 7; 11; 17; 25 µg Cu/L, Fiolen: 0;13; 20; 30; 60; 90 µg Cu/L, St. Envättern: 0; 12; 24; 35; 50; 70 µg Cu/L and Älgsjön: 0; 80; 120; 180; 270; 405 µg Cu/L, in acid cleaned pre-conditioned beakers during a 48 h period. Since the waters differed in DOC concentrations and had different metal background concentrations, the added metal concentrations differed between the studied lakes. For each concentration, 20 daphnids divided into 4 groups of 5 individuals were used. The number of immobile daphnids was recorded at 24- and 48 h. Dissolved oxygen, pH and trace metals (Fe, Al, Zn, Pb, Cd and Cu) were measured at start and end of experiments.

#### Pseudokirchneriella subcapitata

Micro algae tests were performed using a clone of *P. subcapitata*, which has been cultured at Stockholm University since 1975. It is grown in 250 mL culture flasks during constant light in 20 % Z8 culture medium [39]. Growth inhibition by Cu was tested according to OECD [40] during 72 h at three different concentrations for all the lake waters; Abiskojaure: 0, 0.5, 1.5 and 4.5 µg Cu/L, Fiolen and St. Envättern: 0, 5, 15 and 45 µg Cu/L; Älgsjön: 0, 50, 150 and 250 µg Cu/L. However, fluorescence-inferred biomass was used as endpoint instead of dry weight biomass according to modifications in Nyholm [41]. Prior to exposure, glass test tubes (5 mL) were acid cleaned (0.1 M HCl), preconditioned with copper (0–130 µg Cu/L) and enriched with nutrients (50 µg P/L and 5 mg N/L) [41]. Before bioassay start the Z8 medium was replaced with fresh test solution in the test tubes and the algae added. The test tubes (4 concentrations*6 replicates) were held at 20 °C with constant light (60 µE × m^2^ × s^−1^) for 72 h [40]. After 72 h algal density (chlorophyll a 440–460 nM: blue and 685 nM: red) was measured by fluorometry (10-AU Fluorometer Turner Designs).

### Data analysis and BLM

The statistical software PROBIT v. 2.3 was used to calculate bioassay LC- and EC-values as well as their 95 % confidence intervals. Biotic Ligand Model v.2.2.3 [42] was used to calculate LC_50_ results for *D. magna* and *D. pulex*, whereas BLM v 0.0.0.17, based on the Cu-VRA document [43], was used to calculate no observed effect concentration (NOEC) values for the alga and PNEC values for the four lakes. BLM-calculated PNEC values were based on HC_5_ concentration, i.e. where 95 % of test organisms included in the model database will not be affected from the Cu concentration. These HC5 curves are based on species sensitivity distributions (SSDs) for those species that are included in the model’s database [44]. The BLM v.2.2.3 was used since it has a wide calibration range and uses a conversion factor to transform acute to chronic data. The BLM v 0.0.0.17 was chosen since it can predict PNEC values as well as toxicity to *P. subcapitata*. These BLM softwares use the chemical equilibrium model WHAM V [45] to calculate Cu speciation data for LC/EC and PNEC/NOEC values [4, 46]. Neither of these software include DOC origin or the input parameters Fe and Al when calculation Cu toxicity. Differences between modelled and measured toxicity were tested using a one-way ANOVA combined with a post hoc test (Tukey or Dunnett C) (SPSS v. 18). A series of ANCOVAs were applied to ratios between measured and BLM toxicity for *D. magna* and *P. subcapitata*, both species in the same model. The modeled and measured toxicity indices have different definitions for the two species, and hence ratios differ considerably. To obtain approximately equal variances for the two species, the ratios were log-transformed before ANCOVA analyses.

## Abbreviations

ANCOVA: analysis of covariance
ANOVA: analysis of variances
BLM: biotic ligand model
DOM: dissolved organic matter
PNEC: predicted no effect concentration
NOEC: no effect concentration
NOM: natural organic matter
LC: lethal concentration
EC: effect concentration
TOC: total organic carbon

## References

[1] Deleebeeck NME, De Schamphelaere KAC, Janssen CR (2007). A bioavailability model predicting the toxicity of nickel to rainbow trout (*Oncorhynchus mykiss*) and fathead minnow (*Pimephales promelas*) in synthetic and natural waters. Ecotoxicol Environ Saf.

[2] Deleebeeck NME, Muyssen BTA, De Laender F, Janssen CR, De Schamphelaere KAC (2007). Comparison of nickel toxicity to cladocerans in soft versus hard surface waters. Aquat Toxicol.

[3] Paquin PR, Zoltay V, Winfield RP, Wu KB, Mathew R, Santore RC, Di Toro DM (2002). Extension of the biotic ligand model of acute toxicity to a physiologically-based model of the survival time of rainbow trout (*Oncorhynchus mykiss*) exposed to silver. Comp Biochem Physiol C: Toxicol Pharmacol.

[4] Santore RC, Mathew R, Paquin PR, Di Toro D (2002). Application of the biotic ligand model to predicting zinc toxicity to rainbow trout, fathead minnow, and *Daphnia magna*. Comp Biochem Physiol C: Toxicol Pharmacol.

[5] Meyer JS, Santore RC, Bobbitt JP, Debrey LD, Boese CJ, Paquin PR, Allen HE, Bergman HL, Di toro DM (1999). Binding of nickel and copper to fish gills predicts toxicity when water hardness varies, but free-ion activity does not. Environ Sci Technol.

[6] Santore RC, Di Toro DM, Paquin PR, Allen HE, Meyer JS (2001). Biotic ligand model of the acute toxicity of metals. 2. Application to acute copper toxicity in freshwater fish and *Daphnia*. Environ Toxicol Chem.

[7] Kozlova T, Wood CM, McGeer JC (2009). The effect of water chemistry on the acute toxicity of nickel to the cladoceran *Daphnia pulex* and the development of a biotic ligand model. Aquat Toxicol.

[8] Boeckman CJ, Bidwell JR (2006). The effects of temperature, suspended solids, and organic carbon on copper toxicity to two aquatic invertebrates. Water Air Soil Pollut.

[9] De Laender F, De Schamphelaere KAC, Verdonck FAM, Heijerick DG, Van Sprang PA, Vanrolleghem PA, Janssen CR (2005). Simulation of spatial and temporal variability of chronic copper toxicity to *Daphnia magna* and *Pseudokirchneriella subcapitata* in Swedish and British surface waters. Human Ecol Risk Assess.

[10] Bossuyt BTA, De Schamphelaere KAC, Janssen CR (2004). Using the biotic ligand model for predicting the acute sensitivity of Cladoceran dominated communites to copper in natural surface waters. Environ Sci Technol.

[11] Di Toro DM, Allen HE, Bergman HL, Meyer JS, Paquin PR, Santore RC (2001). Biotic ligand model of the acute toxicity of metals: 1. Technical basis. Environ Toxicol Chem.

[12] Meylan S, Behra R, Sigg L (2004). Influence of metal speciation in natural freshwater on bioaccumulation of copper and zinc in periphyton, A microcosm study. Environ Sci Technol.

[13] De Schamphelaere KAC, Heijerick DG, Janssen CR (2003). Refinement and field validation of a biotic ligand model predicting acute copper toxicity to *Daphnia magna*. Comp Biochem Physiol C: Toxicol Pharmacol.

[14] Sciera KL, Isely JJ, Tomasso JR, Klaine SJ (2004). Influence of multiple water-quality characteristics on copper toxicity to fathead minnows (*Pimephales promelas*). Environ Toxicol Chem.

[15] Hoppe S, Gustafsson J-P, Borg H, Breitholtz M (2015). Evaluation of current copper bioavailability tools for soft freshwaters in Sweden. Ecotoxicol Environ Saf.

[16] FOREGS (2011). http://weppi.gtk.fi/publ/foregsatlas/text/Ca.pdf

[17] Wilander A, JohnsonRK, Goedkoop W, Lundin L (1998) Riksinventering 1995. En synoptisk Studie av vattenkemi och bottenfauna i svenska sjöar och vattendrag. Naturvårdsverket, rapport 4813

[18] De Schamphelaere KAC, Vasconcelos FM, Heijerick DG, Tack FMG, Delbeke K, Allen HE, Janssen CR (2003). Development and field validation of a predictive copper toxicity model for the green alga *Pseudokirchneriella subcapitata*. Environ Toxicol Chem.

[19] De Schamphelaere KAC, Janssen CR (2004). Development and field validation of a biotic ligand model predicting chronic copper toxicity to *Daphnia magna*. Environ Toxicol Chem.

[20] Campbell PGC, Stokes PM (1985). Acidification and toxicity of metals to aquatic biota. Can J Fish Aquat Sci.

[21] Andrén CM, Rydin E (2012). Toxicity of inorganic aluminium at spring snowmelt-In-stream bioassays with brown trout (*Salmo trutta* L.). Sci Total Environ.

[22] Jager T (2012). Bad habits die hard: the NOEC’s persistence reflects poorly on ecotoxicology. Environ Toxicol Chem.

[23] Tipping E (1998). Humic ion-binding Model VI: an improved description of the interactions of protons and metal ions with humic substances. Aquat Geochem.

[24] Tipping E, Rey-Castro C, Bryan SE, Hamilton-Taylor J (2002). Al(III) and Fe(III) binding by humic substances in freshwaters, and implications for trace metal speciation. Geochim Cosmochim Acta.

[25] Al-Reasi HA, Wood CM, Smith DS (2011). Physicochemical and spectroscopic properties of natural organic matter (NOM) from various sources and implications for ameliorative effects on metal toxicity to aquatic biota. Aquat Toxicol.

[26] Baken S, Degryse F, Verheyen L, Merckx R, Smolders E (2011). Metal complexation properties of freshwater dissolved organic matter are explained by its aromaticity and by anthropogenic ligands. Environ Sci Technol.

[27] Chappaz A, Curtis J (2013). Integrating empirically dissolved organic matter quality for WHAM VI using the DOM optical properties: a case study of Cu–Al–DOM Interactions. Environ Sci Technol.

[28] Mueller KK, Lofts S, Fortin C, Campbell PGC (2012). Trace metal speciation predictions in natural aquatic systems: incorporation of dissolved organic matter (DOM) spectroscopic quality. Environ Chem.

[29] Tipping E, Lofts S, Sonke JE (2011). Humic Ion-Binding Model VII: a revised parameterisation of cation-binding by humic substances. Environ Chem.

[30] Richards JG, Curtis PJ, Burnison BK, Playle RC (2001). Effects of natural organic matter source on reducing metal toxicity to rainbow trout (*Oncorhynchus mykiss*) and on metal binding to their gills. Environ Toxicol Chem.

[31] Gamble DS, Schnitzer M, Singer PC (1973). The chemistry of fulvic acid and its reactions with metal ions. Trace metals and metal-organic interactions in natural waters.

[32] De Schamphelaere KAC, Janssen CR (2004). Effects of dissolved organic carbon concentration and source, pH, and water hardness on chronic toxicity of copper to *Daphnia magna*. Environ Toxicol Chem.

[33] Andrén C, Rydin E (2009). Which aluminium fractionation method will give true inorganic monomeric Al results in freshwaters (not including colloidal Al)?. J Environ Monit.

[34] Ivarsson H, Jansson M (1993). Regional variation of dissolved organic matter in running waters in central northern Sweden. Hydrobiologia.

[35] Mattsson T, Finér L, Kortelainen P, Sallantus T (2003). Brookwater quality and background leaching from unmanaged forested catchments in Finland. Water Air Soil Pollut.

[36] McKnight DM, Boyer EW, Westerhoff PK, Doran PT, Kulbe T, Andersen DT (2001). Spectrofluorometric characterization of dissolved organic matter for indication of precursor organic material and aromaticity. Limnol Oceanogr.

[37] Persone G, Jenssen C (1994). Third practical training course in aquatic toxicity testing.

[38] OECD (2004) OECD guidelines for the testing of chemicals/section 2: effects on biotic systems, Test No. 202: *Daphnia* sp. Acute Immobilisation Test

[39] SIS (2005) Water quality—freshwater algal growth inhibition test with unicellular green algae (ISO 8692:2004)

[40] OECD (2006) OECD guidelines for the testing of chemicals/section 2: effects on biotic systems, Test No. 201: Alga, Growth Inhibition Test

[41] Nyholm N (1985). Response variable in algal growth-inhibition tests—biomass or growth-rate. Water Res.

[42] Hydroqual (2007) Biotic ligand model version 2.2.3. http://www.hydroqual.com/wr_blm.html

[43] European Copper Institute (2007) Voluntary risk assassment of Copper, Copper II Sulphate Pentahydrate, Copper(I)oxide, Copper(II)oxide, Dicopper chloride trihydroxide, European Union Risk Assessment Report. http://echa.europa.eu/copper-voluntary-risk-assessment-reports

[44] Wheeler JR, Grist EPM, Leung KMY, Morritt D, Crane M (2002). Species sensitivity distributions, data and model choice. Mar Pollut Bull.

[45] Tipping E (1994). WHAM—a chemical-equilibrium model and computer code for waters, sediments, and soils incorporating a discrete site electrostatic model of ion-binding by humic substances. Comput Geosci.

[46] Paquin PR, Gorsuch JW, Apte S, Batley GE, Bowles KC, Campbell PGC, Delos CG, Di Toro DM, Dwyer RL, Galvez F, Gensemer RW, Goss GG, Hogstrand C, Janssen CR, McGeer JC, Naddy RB, Playle RC, Santore RC, Schneider U, Stubblefield WA, Wood CM, Wu KB (2002). The biotic ligand model: a historical overview. Comp Biochem Physiol C: Toxicol Pharmacol.

